# Improving the Infectious Diseases Physician Scientist Workforce From the View of Junior Investigators: Vision, Transparency, and Reproducibility

**DOI:** 10.1093/cid/ciz529

**Published:** 2019-06-20

**Authors:** Jeffrey M Collins, Erika K Wallender, Michael H Woodworth

**Affiliations:** 1 Division of Infectious Diseases, Department of Medicine, Emory University School of Medicine, Atlanta, Georgia; 2 Division of HIV, Infectious Diseases, and Global Medicine, Department of Medicine, University of California, San Francisco

**Keywords:** graduate medical education, clinical research, infectious disease

## Abstract

Shortcomings in the current pipeline of infectious disease physician scientists are well documented. With a focus on the transition of early stage investigators to research independence, we outline challenges in existing training pathways for physician scientists. We urge leaders of infectious disease societies, divisions, and governmental and nongovernmental funding organizations to reinvigorate a vision for nurturing trainees with interests in research, to seek transparency in physician scientist funding mechanisms, and to encourage efforts to improve the reproducibility of outcomes for talented junior investigators. We feel that the alternative to making these changes will lead to further drop-off in the physician scientist pipeline in a field that has a perpetual need for research.

## Teaser

Three junior aspiring physician scientists outline challenges in establishing research independence and respond with recommendations to improve the pathway.

## THE PERPETUAL NEED FOR INNOVATION IN INFECTIOUS DISEASES

In 2012, Dr Anthony Fauci, the director of the National Institute of Allergy and Infectious Diseases (NIAID), described the perpetual challenges of infectious diseases (ID) and wrote that “our response must be perpetual as well” [1]. Since these words were published, the world has seen its largest outbreak of Ebola virus disease, the emergence of pan-resistant gram negative bacteria, and multidrug resistant *Candida auris,* and a generation of children and their families now face the life-long consequences of congenital Zika virus infection [2–6]. These crises exemplify our perpetual need for ID research to understand pathogenesis, diagnosis, treatment, and prevention of emerging and recognized infectious diseases. Bearing this in mind, thought leaders recently outlined the need to optimize the ID physician-scientist workforce, which is rapidly aging and not being adequately replaced by junior investigators [7]. Clearly, the perpetual need for ID research is closely linked to a perpetual need to provide new investigators the protected time and mentorship necessary to meet these challenges.

As 3 junior investigators with sustained interests in becoming physician-scientists, we have found the transition from ID fellowship to junior faculty to be one of the greatest barriers to entering a research career. We outline the challenges that ID fellows face when training to become physician-scientists and call for concrete policy initiatives to enhance the vision, transparency, and reproducibility of available training mechanisms. Failing to clarify and standardize the expectations and mentorship pathways for developing physician-scientists will inject increasing doubt in the calculus of trainees considering a future in academic ID at a time when some have questioned the vitality of the field [7]. We argue that without improving these critical areas, the ultimate impact will be a net loss to society in our capacity to address the challenges so well outlined by Dr Fauci in 2012.

## THE CURRENT TRAINING ENVIRONMENT FOR THE PHYSICIAN-SCIENTIST

### Limited Research Experience Before Fellowship

The inherent challenge for early stage physician-scientists is the need for rigorous training to achieve independence as both a clinician and a researcher. For the typical trainee, ID fellowship is the first opportunity for continuous research training and mentorship. Research conducted during standard medical school and residency programs is generally limited to a few discontinuous months, over at least 7 years, potentially across multiple institutions, and may lack rigorous training in scientific methods. These prefellowship experiences are often insufficient to gain the skills and publication track record necessary to compete for extramural funding. Even individuals who take time away from their clinical training to pursue additional research training (through a PhD or other research-intensive training program) usually spend 4 or more years away from their research careers to complete the clinical training. During these interrupted periods of dedicated research time, advances in the field and limited time to produce preliminary data also make it challenging to compete for extramural funding immediately after completing clinical training. As a result, most trainees entering an ID fellowship require a significant amount of protected research time to establish or renew research interests and generate a publication record that would garner favorable review.

### Scarce Funding for Research During Fellowship

Funding options for protected research time during ID fellowship are not available for all fellows interested in a physician scientist career. The first 2 years of ID fellowship are often funded through graduate medical education (GME) sources, which require clinical effort. A minority of programs can offer fellows a third year of protected research time in the absence of an external funding source.

In the United States, the most common funding mechanism for research training is the National Institutes of Health (NIH) T32 National Research Service Award (NRSA) fellowship, which typically funds 2 years of research training. However, these grants are awarded to specific institutions and are generally tied to specific areas of research. If a trainee’s institution does not have a T32 or their research falls outside the scope of an institution’s T32, the options for fellowship research funding quickly diminish.

**Figure 1.**
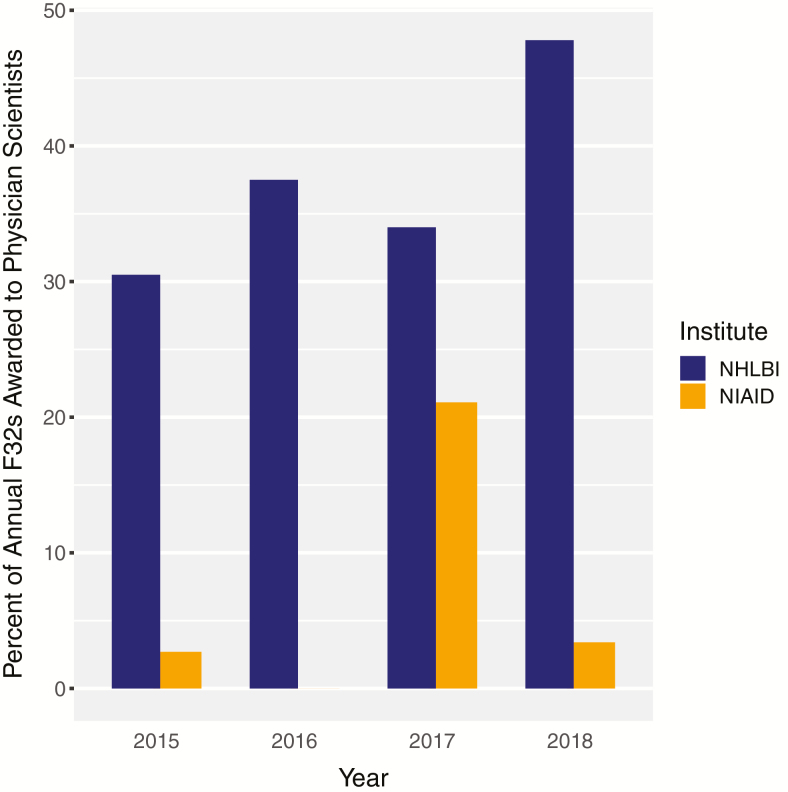
Percentage of F32 awards from the NIAID and the NHLBI supporting physician scientists from 2015 to 2018. The number of NHLBI F32 grants awarded to physician scientists was 18/59 (30.5%), 24/64 (37.5%), 18/53 (34%), and 33/69 (47.8%) in 2015, 2016, 2017, and 2018, respectively. During the same years the number of NIAID grants awarded to physician scientists was 1/37 (2.7%), 0/36, 4/19 (21.1%), and 1/29 (3.4%). Abbreviations: NHLBI, National Heart, Lung and Blood Institute; NIAID, National Institute of Allergy and Infectious Diseases.

If a T32 position is not available, alternative sources of postgraduate research funding include the F32, U-series, K99/00 awards, and foundation or industry postdoctoral training awards. The F32 is a nationally competed individual NRSA grant that is not tied to institutions. Although this is an attractive option, when we dual-extracted data from NIH RePORTER and publicly available fellowship and social networking websites, we identified only 2 trainees affiliated with an ID fellowship program and 6 total physician scientists have been supported by an NIAID F32 award from 2015 to 2018. These awards accounted for 1.7% and 5.0% of total awards during that period respectively (Figure 1) [8]. This is a stark contrast to the National Heart, Lung, and Blood Institute, where 38.0% of F32s were awarded to physician scientists in training over the same period. Although the number of ID fellows applying for these awards is not publicly available, these data suggest physician scientists in training either have extremely low success rates or do not view this as a viable funding mechanism. A handful of foundation and multicenter U-series grants such as the Antibacterial Resistance Leadership Group (ARLG) exist, but these are competitive awards that support a small number of fellows. The more recently introduced NIH K99/R00 award funds 2 years of research training at the postdoctoral level before requiring recipients to transition to research independence [9]. However, most physician scientists need more than 2 years of mentored research support, and none of the NIAID K99/R00 awardees from 2015 to 2018 completed an ID fellowship [8]. Again, the number of physician scientists competing for this award is not known, but the outcomes suggest this pathway is of limited relevance for persons completing clinical training programs who often do not yet possess extensive research experience and publication records.

### A Competitive Transition to Junior Faculty

**Figure 2.**
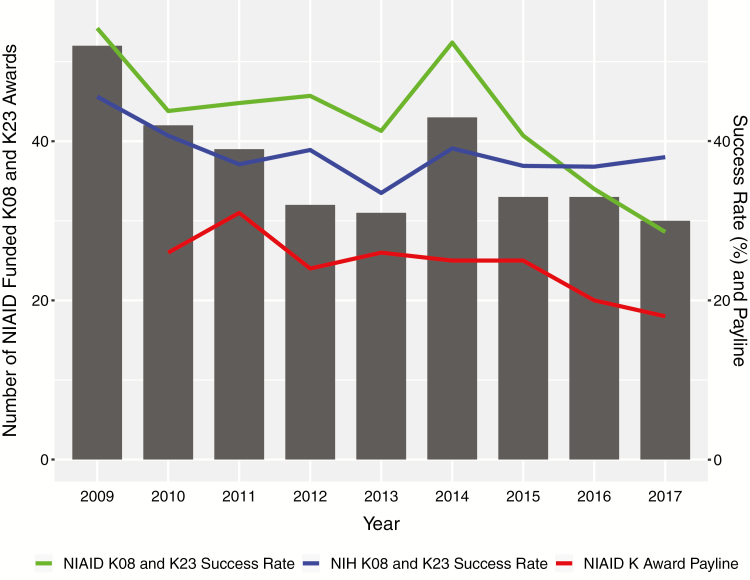
Funded NIAID K applications, funding rates, and paylines 2009–2017. The total number of K08 and K23 awards each year is represented by the bar graph. The line graphs depict the NIAID combined K08 and K23 success rate (green), the NIH-wide combined K08 and K23 success rate (blue) and the NIAID K award payline (red). Abbreviations: NIAID, National Institute of Allergy and Infectious Diseases; NIH, National Institutes of Health.

The dual training demands required of ID physician scientists make NIH career development awards a key strength and growing necessity to transition to junior faculty. NIH K-series awards give physician investigators up to 5 years of protected research time to further develop their research programs and transition to independence. Unfortunately, although the number of NIAID K23 and K08 applications nearly matched a 10-year high in 2017, the number of awards has declined by over 40% from 54 in 2008 to 30 in 2017 (Figure 2). Concerns over such trends were amplified after the interim payline for FY19 was initially set at 14 before being revised to 20 [10]. The available data suggest that lower paylines are the result of a growing gap between the number of applications and the number of funded awards [11].

K awards often form the basis for academic appointments for ID physician scientists, and shrinking this funding mechanism acutely impacts early investigators at a critical stage in their career [12]. In many cases, failure to obtain a career development award leaves physician scientists with the option of either leaving scientific research or delaying an academic faculty appointment until a K award is obtained. Those who choose to delay seeking an academic faculty appointment generally have 2 pathways that allow them to pursue the mentored clinical research training they need: the “nights and weekends” model or the “perpetual ID fellow” model.

Under the “nights and weekends” model, the aspiring physician scientist accepts a primarily clinical academic faculty appointment that commits to 50–100% of their effort to clinical work. Continuing research necessary to gain extramural funding is voluntary and not compensated. At a life stage associated with increasing demands in one’s personal life such as buying a home or having children, this is a challenging prospect. Further, ID consult service volume appears to be rising with increasing documentation requirements and patient acuity across the nation [13], making it much more challenging to progress toward research goals without protected research time.

The “perpetual fellow” typically accepts a nonacademic appointment as an instructor or similar level position. In this model, research remains voluntary and uncompensated, but less clinical time is required to fund the lower salary of an instructor leaving more time to develop one’s research interests. Although the personal strain of having to do research, write papers, and develop grants in one’s free time is relieved, it is replaced by financial strain at a time when medical student debt continues to rise, and many are struggling with the added costs of starting a family [14]. Although programs such as the NIH Loan Repayment Program can offset some of the opportunity costs of lower salaries, many top infectious disease training programs (which also are frequently affiliated with T32 grants and sought-after research mentors) are located in cities with high costs of living, which may require trainees and junior faculty to moonlight to offset the financial challenges of deferring faculty appointment.

Neither of these 2 pathways necessarily identifies the most talented scientists, nor do they provide sufficient resources to adequately nurture those in need of research career development. Further, setting these paths as the benchmark for normal career development is a potential deterrent for trainees considering a career as an ID physician scientist.

## RESEARCH TRAINING AS A LONG-TERM INVESTMENT

**Figure 3.**
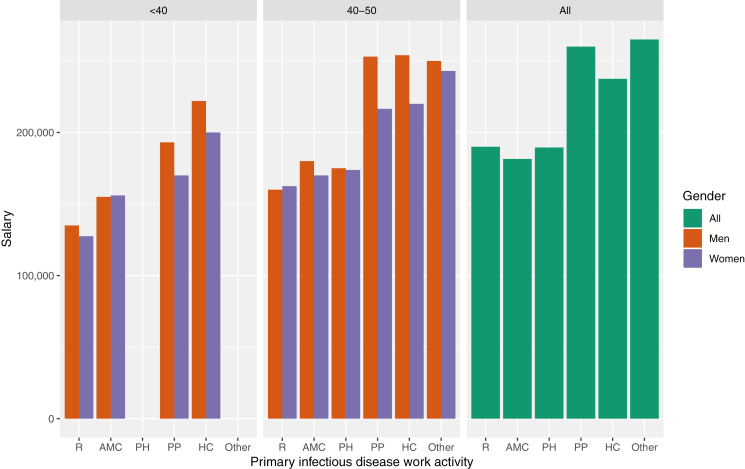
Median salary in US dollars of infectious disease career paths by age group, gender, and primary work activity among respondents to the 2017 Infectious Disease Society of America member compensation survey. Note that salary data not reported for public health and “other” categories in the <40 age group due to low response rates in these categories. Abbreviations: AMC, academic medical center clinical position; HC, hospital/clinic clinical position; PH, public health; PP, private practice clinical position, R, primary research position.

We have been highly motivated by the satisfaction of scientific inquiry and opportunity for public health impact provided by a research career. However, it is also important to consider the impact of further financial disincentives borne disproportionately by ID physician scientists. A compensation survey commissioned by the Infectious Diseases Society of America in 2017 showed the median salary for those at academic medical centers was markedly lower compared to their counterparts in private practice or public health (Figure 3) [15]. Within academic medical centers, early career physician scientists appear to pay a steeper price. Young male physician-scientists earn a median of $20 000 less than young male academic ID clinical faculty. For women, the difference is $30 000. These differences accentuate the financial sacrifices involved in extending fellowship programs to gain research training and develop a publication record at a life stage associated with compounding economic stresses. Although a substantial number of trainees are willing to make financial sacrifices to pursue careers as ID physician scientists, we argue that concentrating these penalties among early career physicians, at a time of decreasing funding security, could serve as a particularly strong disincentive for early stage investigators.

## RECOMMENDATIONS FOR OUR LEADERS TO SUPPORT JUNIOR PHYSICIAN-SCIENTISTS

### Vision

When considering ways to expand the ID physician scientist workforce, the transition from trainee to mentored physician scientist is a critical step. We recommend institutions lay out a renewed vision of explicit pathways for a trainee to transition from fellow to mentored faculty (Table 1).

**Table 1.** Challenges for Infectious Disease Physician Scientist Career Development and Recommendations to Address Them

Challenge	Recommendation
• Completing clinical training is important but reduces opportunities for early career publications which are necessary to win independent funding.	• Expand funding experimentation with programs like the R38 series. • Adding funding support for certificate or degree programs in research for these early postdoctoral physician trainees.
• Research funding for protected time during ID fellowship is scarce.	• Review physician postgraduate funding rates for F32 fellowships and expand if indicated. • Expand programs like the ARLG fellowship that are open to application across institutions. • Reduce wait times between K-series application submission and committee evaluation. • The IDSA foundation could consider enhanced private-sector engagement to expand IDSA-administered fellowship grants
• Messages about need for more ID research and physician scientists conflicts with low NIAID K award paylines.	• Commission a needs assessment through IDSA or other mechanisms for the ID physician scientist career development pipeline. • Expand K series career development funding if more research and researchers are needed.
• Benchmarks for K award candidate evaluation may be obscure.	• Improve public reporting of interquartile ranges of number of publications of successful K award applicants and describe expectations for mentorship and institutional support. • Increase transparency about funding priorities for NIAID.
• Limited funding shifts burden onto trainees to build research portfolio through “nights and weekends” and “perpetual fellow” models.	• Expand early career funding like KL2 and ARLG Early Stage Investigator grants to support research capacity and publication record prior to K application. • IDSA should seek to develop new career development awards through private or foundation donation support.

Abbreviations: ARLG, Antibacterial Resistance Leadership Group; ID, infectious disease; IDSA, Infectious Disease Society of America; NIAID, National Institute of Allergy and Infectious Diseases.

Efforts by NIAID to experiment with novel funding pathways designed for physician scientists, such as the R38 Stimulating Access to Research in Residency (StARR) program should be applauded [16]. This program protects the time of physician scientists throughout their residency programs to provide a more substantive research experience of up to 2 years. StARR could be further enhanced by funding certificate or degree programs in research that are not current features of all of these programs. Graduates of the R38 program can then apply for a nationally competed K38 award, which protects up to an additional 2 years of research time for awardees during the medical fellowship program of their choice. Completion makes immediate competition for career development awards (K23 or K08) or pathway to independence awards (K99/R00) more feasible for medical trainees. The recently announced physician-scientist K99/R00 award is another important NIH response to this group’s low K99/R00 funding success and the impact of this new mechanism should be assessed [17].

However, until such programs can be refined and expanded, there is a substantial need for additional nationally competed research training grants like the ARLG awards that allow aspiring physician scientists 2 years of protected time for mentored research training during fellowship. Although the ARLG fellowship relies on established project funding and mentorship, it provides unique opportunities in clinical and translational research exposure, expanded networking with national leaders in a priority area of research, and enhanced mentorship and support for application to NIAID career development awards [18].

We encourage our professional societies to conduct needs assessments of the research work force, and if supported by these findings, to seek additional private, foundation, and federal funding for early career development of physician scientists. The F32 NRSA fellowship program should be reviewed to determine why aspiring physician scientists are either not pursuing or not receiving these research awards. Although institutional T32 grants provide valuable training support in specific fields, it is essential that physician scientists have some opportunity for research training in the event that their fellowship institution does not have a T32, or the award falls outside their research interests.

There must also be a vision for how these fellowship programs will lead to mentored clinical research awards and the timeline for completing the transition. A dedicated 2-year research fellowship is often not enough time for a trainee without significant research experience to develop a publication record competitive for a mentored career development award. For those who are able to submit a competitive application during fellowship, most will require at least 1 resubmission. Thus, we believe novel funding mechanisms are needed to support physician scientists during this transition. One potential solution would be expansion of 1–2 year foundation or institution grants that could be used to protect research time for promising physician scientists during this transitional period. The 3-year career development awards offered by the American Heart Association or Doris Duke Foundation, and the 2-year PhRMA Foundation Faculty Development Award could serve as a model for such programs [19–21].

Finally, streamlined mechanisms for physician scientist development must be accompanied by a larger pool of career development awards to which physician scientists can apply. Without this, increasing the number of qualified applicants for career development awards may further drive down success rates, while having a negligible effect on the physician scientist workforce. We hope the recent increase in the NIAID K award payline signals renewed investment in this area.

### Transparency and Timeliness

We feel that improving transparency and reducing wait times for early career grant funding decisions could improve the ID physician scientist pipeline. Unspoken priorities from application reviewers include an expected number of first author publications, concrete types of mentorship and division or department commitment, an institute’s current research area priorities, or shifts in internal funding distribution that changes the number of awards available. A lack of transparency can derail years of planning for the trainee, introduce bias, and undercut the value of mentorship if the formulae for successful applications are also not apparent to mentors or division chiefs [22].

We suggest leadership at NIAID report the interquartile ranges of first and coauthor publications associated with fundable K application scores, compared to nonfundable scores and triaged results. As publications for preclinical or basic science research can be more time-intensive, a goal number of publications can inform planning for a more diverse profile of publications that may be more feasible to accomplish in a limited period of time.

We also suggest improved public discussion of priority content areas and funding mechanisms to allow trainees an opportunity to pivot in their training and productivity goals. If trainees are not given enough time to respond to shifts in funding priorities related to areas of investigation or career pathways, this can lead to unnecessary attrition of talented young investigators. The vision for training the next generation of physician scientists must evolve with the field, and as it evolves, that vision must be clearly communicated to those pursuing that career pathway.

Finally, with limited available research funding during fellowship and a transition to faculty increasing tied to NIH funding, we suggest that NIAID prioritize a more rapid review of K-series career development awards. As has become precedent for review of proposed studies about human immunodeficiency virus/AIDS [23], incentivizing more rapid decisions for K-series awards will allow trainees to efficiently plan their future, either by allowing them to apply for additional short term funding, resubmit within less than 1 year of the initial submission, or plan for their employment while still protected by fellowship funding.

### Reproducibility

We believe that enhancing the reproducibility of funding success for aspiring physician scientists will improve the quality of applications as well as the equity of funding decisions. A successful transition from fellow to academic physician scientist currently depends on the alignment of a variety of circumstantial factors including location of training, available mentorship, capacity of the applicant’s academic institution to support trainees, and the NIH peer review process. However, we are not aware of a coordinated effort to understand the proportional impact of these factors on physician scientist funding success. We applaud recent efforts by the NIH to increase the representation of women and underrepresented minorities in grant funding. As a next step, we recommend that ID fellowship programs and the NIH prospectively study the factors associated with junior physician scientist funding success. These data could inform an evidence-based approach to mentorship that could aid division directors and mentors in addressing shortcomings and improve resource allocation to support the reproducibility of funding success. Understanding existing barriers to physician scientist career development could aid in the encouragement and retention of candidates who are creative, diverse, and successful—keys to strengthening the ID physician scientist work force. We argue that supporting the perpetual evaluation of who succeeds in becoming physician scientists, who does not succeed, and how we can improve the reproducibility of our system will greatly support the vitality of our community of ID physician scientists.

## CONCLUSION

As demonstrated by increasing numbers of trainees and junior investigators applying for mentored scientist awards from NIAID, trainees are responding to the call for increasing the ID physician scientist work force. However, without a clear and transparent vision as to how this interest will be translated into higher numbers of independent physician investigators, we believe there is risk of driving talented physician scientists away from ID. We urge our ID leaders to develop a vision for explicit mechanisms for developing ID trainees into independent physician scientists, seek transparency at NIAID and other agencies regarding pathways that fund physician scientists, and encourage efforts to improve reproducibility of outcomes for talented junior investigators. We feel that the alternative to making these changes will be a net loss to society.

## Notes

***Acknowledgments.*** The authors thank Dr Wendy Armstrong for encouragement and advocacy in preparing this manuscript.

***Financial support.*** M. H. W. was supported in part by an Antibacterial Resistance Leadership Group fellowship [National Institute of Allergy and Infectious Diseases UM1AI104681]. E. K. W. was supported in part by the Clinical Pharmacology, Drug Action and Pharmacogenetics T32 [National Institute of General Medical Sciences T32GM007546].

***Potential conflicts of interest.*** The authors: No reported conflicts of interest. All authors have submitted the ICMJE Form for Disclosure of Potential Conflicts of Interest. Conflicts that the editors consider relevant to the content of the manuscript have been disclosed.
